# Analysis of Free Energy Signals Arising from Nucleotide Hybridization Between rRNA and mRNA Sequences during Translation in Eubacteria

**DOI:** 10.1155/BSB/2006/23613

**Published:** 2006-11-29

**Authors:** Lalit Ponnala, Anne-Marie Stomp, Donald L Bitzer, Mladen A Vouk

**Affiliations:** 1 Department of Electrical and Computer Engineering, North Carolina State University, Raleigh, NC 27695, USA; 2 Department of Forestry, North Carolina State University, Raleigh, NC 27695, USA; 3 Department of Computer Science, North Carolina State University, Raleigh, NC 27695, USA

## Abstract

A decoding algorithm is tested that mechanistically models the progressive alignments that arise as the mRNA moves past the rRNA tail during translation elongation. Each of these alignments provides an opportunity for hybridization between the single-stranded, -terminal nucleotides of the 16S rRNA and the spatially accessible window of mRNA sequence, from which a free energy value can be calculated. Using this algorithm we show that a periodic, energetic pattern of frequency 1/3 is revealed. This periodic signal exists in the majority of coding regions of eubacterial genes, but not in the non-coding regions encoding the 16S and 23S rRNAs. Signal analysis reveals that the population of coding regions of each bacterial species has a mean phase that is correlated in a statistically significant way with species () content. These results suggest that the periodic signal could function as a synchronization signal for the maintenance of reading frame and that codon usage provides a mechanism for manipulation of signal phase.

## 

[1,2,3,4,5,6,7,8,9,10,11,12,13,14,15,16,17,18,19,20,21,22,23,24,25,26,27,28,29,30,31,32]
